# Edge-Computing Video Analytics Solution for Automated Plastic-Bag Contamination Detection: A Case from Remondis

**DOI:** 10.3390/s22207821

**Published:** 2022-10-14

**Authors:** Umair Iqbal, Johan Barthelemy, Pascal Perez, Tim Davies

**Affiliations:** 1 SMART Infrastructure Facility, University of Wollongong, Wollongong, NSW 2522, Australia; 2 NVIDIA, Santa Clara, CA 95051, USA

**Keywords:** waste contamination, edge-computing, Artificial Intelligence of Things (AIoT), computer vision, deep learning

## Abstract

The increased global waste generation rates over the last few decades have made the waste management task a significant problem. One of the potential approaches adopted globally is to recycle a significant portion of generated waste. However, the contamination of recyclable waste has been a major problem in this context and causes almost 75% of recyclable waste to be unusable. For sustainable development, efficient management and recycling of waste are of huge importance. To reduce the waste contamination rates, conventionally, a manual bin-tagging approach is adopted; however, this is inefficient and requires huge labor effort. Within household waste contamination, plastic bags have been found to be one of the main contaminants. Towards automating the process of plastic-bag contamination detection, this paper proposes an edge-computing video analytics solution using the latest Artificial Intelligence (AI), Artificial Intelligence of Things (AIoT) and computer vision technologies. The proposed system is based on the idea of capturing video of waste from the truck hopper, processing it using edge-computing hardware to detect plastic-bag contamination and storing the contamination-related information for further analysis. Faster R-CNN and You Only Look Once version 4 (YOLOv4) deep learning model variants are trained using the Remondis Contamination Dataset (RCD) developed from Remondis manual tagging historical records. The overall system was evaluated in terms of software and hardware performance using standard evaluation measures (i.e., training performance, testing performance, Frames Per Second (FPS), system usage, power consumption). From the detailed analysis, YOLOv4 with CSPDarkNet_tiny was identified as a suitable candidate with a Mean Average Precision (mAP) of 63% and FPS of 24.8 with NVIDIA Jetson TX2 hardware. The data collected from the deployment of edge-computing hardware on waste collection trucks was used to retrain the models and improved performance in terms of mAP, False Positives (FPs), False Negatives (FNs) and True Positives (TPs) was achieved for the retrained YOLOv4 with CSPDarkNet_tiny backbone model. A detailed cost analysis of the proposed system is also provided for stakeholders and policy makers.

## 1. Introduction

The waste generation rate is reported to have increased in the last couple of decades mainly because of the increase in economic development and urbanization [1,2]. Increased waste volumes are causing problems for governments in managing and processing them efficiently [3,4]. Although developed countries have proper waste classification systems in place (i.e., red, green, yellow), most of the waste still ends up either in landfills or incinerated, mainly because of the presence of contamination (Ziouzios et al. [5] suggests that 75% of the municipal waste that may be recycled is wasted). Therefore, it is of significant importance for any country to enhance its ability to improve waste recycling and waste management mechanisms. Both the existing waste management techniques of landfilling and incinerating pose serious environmental and health threats to the community [3,6,7,8].

In the context of sustainable development, efficient waste management is one of the key agendas which directly influences the sustainable development goals (SGDs) [9,10]. However, irrespective of its importance in global sustainability, waste management has been less prioritized compared to other factors such as water and energy. Specifically, in the context of Australia, limited resources are allocated for waste management (e.g., 250 million AUD were allocated for the waste recycling and policy action plan [9]). The Chinese waste ban in 2018 and the Council of Australian Government (COAG) export ban in 2020 have caused a national waste crisis. As of now, local governments are individually responsible for the management of waste (i.e., collection, disposal, recycling).

At the scale of local waste management, contamination in household waste is one of the highlighted challenges that significantly impacts the waste recycling process [11]. As a standard, within Australia, a 6% to 10% contamination rate is referred to as an acceptable range; however, in recent times, the average contamination rates have been reported to be around 15%, which is much higher than the recycling waste import threshold of only 0.5% imposed by China. Educating the community through various activities, workshops and webinars is reported to be one of the commonly suggested approaches towards reducing household contamination. However, such an initiative may only be successful if accurate and widespread data is shared with the community to motivate them [12,13].

At the local government scale, bin-tagging or waste auditing is the adopted approach to collect waste contamination-related data and to report the contamination to corresponding customers [9]. However, bin-tagging is done mainly by the waste collection truck driver by manually looking into the waste truck hopper captured by the camera [9,14]. Remondis is the leading waste management organization within the Illawarra, New South Wales (NSW), Australia, and has camera-based systems installed to facilitate the manual bin-tagging process. However, manual bin-tagging is a labor-intensive process that also impacts the driving capabilities of waste collection truck drivers. Manual data collection involves the subjective visual observations of the driver, which may result in high data variance, which needs further analysis and time resources. Therefore, there is a dire need for a unified automated waste contamination detection system using the state-of-the-art technologies towards efficient and sustainable waste management. In this context, detection of plastic-bag contamination, which is one of the most common forms of contamination in household waste, is considered as the first step for developing an automated system.

Artificial Intelligence (AI), edge-computing, Artificial Intelligence of Things (AIoT), computer vision and the Internet of Things (IoT) are disruptive technologies that have achieved huge success in dealing with complex real-world problems [15,16,17,18,19]. In the context of waste management, various studies have been performed for waste detection and classification [5,20,21,22,23,24,25,26,27,28,29]; however, there still exists a gap in the development of a practical solution. This paper presents an edge-computing video analytics solution for automated plastic-bag contamination detection to be used in waste collection trucks for efficient contamination detection. The proposed solution implements the state-of-the-art object detection algorithms to detect plastic-bag contamination in household waste. Multiple variants of the Faster R-CNN and You Only Look Once version 4 (YOLOv4) models have been trained for plastic-bag contamination detection. For training the computer vision models, a real utility-oriented dataset (i.e., Remondis Contamination Dataset (RCD)) was developed from the manually tagged records of the Remondis collection trucks. The following are the anticipated contributions of the presented research:

1. Development of a challenging utility-oriented waste contamination dataset (i.e., RCD) from the Remondis manual bin-tagging historical records and annotation for plastic-bag contamination bboxes;
2. Development, validation, and analysis of an edge-computing practical solution for automated plastic-bag contamination detection in waste collection trucks.

The rest of the article is organized as follows. Section 2 presents a review of the most relevant benchmark literature related to the use of computer vision technologies for waste detection and classification. Section 3 provides details about the dataset used for the training and validation of the computer vision models. Section 4 presents details about the proposed automated plastic-bag contamination detection system including the software and hardware components. Section 5 provides information about the experimental protocols and evaluation measures. Section 6 details the software and hardware evaluation results for the proposed system, mainly for the computer vision models. Section 7 discusses the results and highlights the potential challenges of the problem. Section 8 presents information about the field data collection and retraining of the model for improved performance as an essential step from an enterprise solution development perspective to ensure admissible field performance. Section 9 provides detailed cost analysis for the proposed plastic-bag contamination detection system. Finally, Section 10 concludes the study by highlighting the important insights and listing potential future research directions.

## 2. Related Work

This section presents a review of benchmark literature in regards to waste detection and classification using computer vision and edge-computing technologies. The review is organized in chronological order to highlight the advancements made over time in the domain of waste detection.

Rad et al. [20], in the year 2017, proposed a computer vision-based litter localization and classification system using the OverFeatGoogleNet model. A custom-collected dataset of around 4000 images was used to train the computer vision models. From the results, the proposed approach was able to achieve a detection precision of 63%. The detected litter objects (e.g., leaves, cigarette buts) are not directly related to the waste contamination; however, the detection of small litter objects from the image makes it a relevant problem from a computer vision perspective. Ibrahim et al. [21], in 2019, developed a comprehensive waste-contamination dataset (i.e., ContamiNet) towards detecting contamination in solid waste. The dataset consists of 30,000 images from multiple sources where the contamination was identified within the waste. The CNN model was trained and compared against the manual labeling. The trained CNN model was able to achieve an AUC of 0.86 compared to the manual AUC of 0.88.

Kumar et al. [22], in 2020, proposed the use of a computer vision object detection model (i.e., YOLOv3) for efficient waste classification. A custom-developed dataset of approximately 8000 images of waste from six different classes was used to train the object detection model. Most of the images in the dataset contained a single object belonging to only one class; however, a few test images were also captured from the real world where multiple objects belonging to multiple classes were present. From the experimental investigations, an mAP of 95% was achieved by the YOLOv3 model. Later in the same year, Li et al. [23] proposed a YOLOv3-based computer vision solution to detect water surface garbage. A custom-developed dataset of 1200 images was used to train the waste detection model. From the experimental analysis, the proposed YOLOv3 model was able to achieve an mAP of 91% among three garbage classes (i.e., bottle, plastic, Styrofoam). Although high detection performance was reported, the dataset used for the training was not challenging enough and involved very minimal background noise because the presence of water made the waste objects distinct for the detector.

Panwar et al. [24], in 2020, proposed a dataset called AcquaVision to facilitate the use of deep transfer learning toward detecting waste objects in water. The dataset comprised 369 images annotated for four waste categories (i.e., glass, metal, paper, plastic). The RetinaNet model was implemented to detect the waste objects from images and reported an mAP of 81%. Although the implemented model performed well, the dataset was very limited and the reported performance cannot be considered a generalized performance. The images from the dataset were of good resolution with distinct waste objects and no noise in most cases (i.e., only the waste objects were present in the image). The similarity between paper and plastic bags is one of the challenges to address in this case. White et al. [25], in 2020, developed a novel CNN model referred to as WasteNet toward classifying waste objects in the context of smart bins. The proposed model was based on the VGG16 transfer learned architecture and was trained using the TrashNet dataset consisting of 2500 images classified across six different trash classes. From the results, the proposed WasteNet model was able to achieve 97% prediction accuracy. Although high performance of the proposed model was reported, it was not compared to other literature where the TrashNet dataset was used. Further, the nature of the dataset was not complex and the image consisted of only a single class object without any noise, making it a simpler problem for a CNN-based classifier.

Kraft et al. [26], 2021, developed an edge-computing solution for unmanned aerial vehicles to detect trash from low altitudes. An NVIDIA Jetson NX edge-computer with an object detection model was used to detect the small trash objects from the air. The computer vision models were trained using the UAVVaste dataset consisting of 774 images with 3716 bbox annotations of trash. YOLOv4, EfficientDet and Single Shot Detector (SSD) computer vision object detection models were trained and compared for their performance. From the results, the YOLOv4 model was able to achieve mAP@50 of 78%. Patel et al. [27], in 2021, used multiple computer vision object detection models to detect garbage. The dataset consisted of 544 images with bbox annotations of garbage material in the image. EfficientDet, RetinaNet, CenterNet and YOLOv5 models were trained and performance was compared. From the experimental analysis, the YOLOv5 model was able to achieve an mAP of 61%. The dataset used was very limited and reported performance cannot be considered a generalized performance; however, the images in the dataset were from challenging real-world scenarios.

Chazhoor et al. [28], in 2022, performed a comprehensive benchmark study to classify plastic waste using CNN transfer learned models. The WaDaBa dataset consisting of around 4000 images from seven different plastic waste classes was used to train the CNN models (i.e., AlexNet, ResNet50, ResNeXt, MobileNetv2, DenseNet, SqueezeNet). From the experimental analysis, ResNeXt was reported to perform best with an AUC of 94.8%. High classification performance was reported for CNN transfer learned models, which may be attributed to the noise-free dataset. Furthermore, results were not discussed in line with the literature where the WaDaBa dataset was already used. Radzi et al. [29], in 2022, proposed the use of CNN classification models (e.g., ResNet50) to classify given plastic waste images into seven classes (i.e., PET, HDPE, PVC, LDPE, PP, PS, others). A custom-developed dataset of 2110 images was developed and manually annotated for seven different classes of the plastic type. From the results, the ResNet50 model was able to achieve a classification accuracy of 94%. Although the implemented model was able to achieve high accuracy, the dataset was very simple, consisting of cropped images of individual plastic objects (i.e., only one class of object in a single image), which is not the case in most practical applications, where such models are prone to fail drastically. Most recently, Ziouzios et al. [5] developed a real-time waste-detection and classification system towards efficient solid waste management. The dataset used for the training of models consisted of 1500 images from the TACO dataset and 2500 images from a local waste-treatment agency belonging to four waste classes (i.e., plastic, glass, aluminum, other). YOLOv4 with CSPDarkNet backbone was trained and reported to achieve an mAP of 92%. Although the reported accuracy is towards the higher end, the images from the TACO dataset are not very challenging and are anticipated to be the reason for the higher accuracy.

As a summary of the literature review (see Table 1), the waste detection problem has been reported to be addressed either as an image classification problem or as an object detection problem. However, the computer vision object detection approach for detecting waste objects is more suitable for the real-world scenario. OverFeatGoogleNet, CNN, WasteNet, AlexNet, ResNeXt, ResNet50, DenseNet, SqueezeNet and MobileNet models are the highlighted image classification models used in the literature, while YOLOv3, YOLOv4, YOLOv5, RetinaNet, EfficientDet, CenterNet and SSD are the highlighted object detection models. In most of the cases, the datasets were either not comprehensive or not challenging enough (i.e., single object per image with no background noise). These critical analyses clearly suggested the need to develop a practical solution with challenging real-world data towards identifying contamination within solid waste.

## 3. Remondis Contamination Dataset (RCD)

The Remondis Contamination Dataset (RCD) used for the development of computer vision models (i.e., training, testing) was established from the historical records of Remondis where the drivers manually labeled the images as contaminated. All the images are stored in jpeg format with 640×480 dimensions and 72 pixels-per-inch resolution. The color scheme for all the images is RGB. The images are taken from the camera installed on the waste collection truck, pointing towards the truck hopper where waste is emptied from the bins before being processed to the main compartment. A portion of images were also captured from the camera pointing towards the bins. The images in the dataset are diverse in terms of at least three different camera zooms, offer challenging blur noise and are captured from different angles depending on the settings of camera installed on the truck. The dataset presents various waste contaminants including plastic bags, plastic bottles and food waste. RCD is a novel dataset presented for the first time in this manuscript and can serve as a benchmark for practical waste segregation purposes including detection of different waste contaminants, characterization of waste contents and counting of a certain waste content occurrence. The main differences between the existing waste contamination datasets and RCD are the actual real-world visuals and presence of contamination along with the non-contaminated waste. For the presented research, the raw dataset was labeled to detect plastic-bag contamination only.

**Figure 1:**
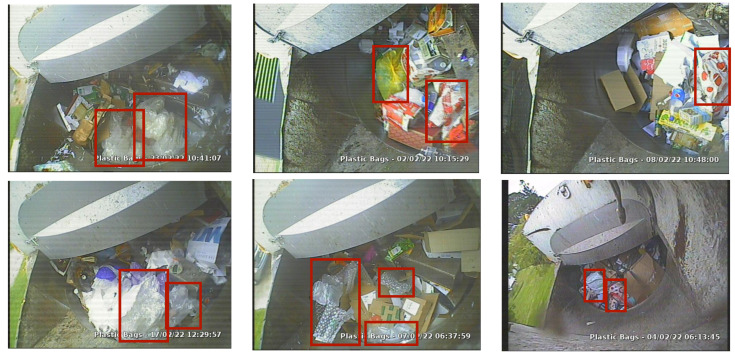
Annotated samples from the RCD.

In terms of plastic waste contamination, the dataset is highly challenging, mainly because of visual similarities between some types of plastic bags and non-contaminants. For example, a white plastic-bag is often similar to white paper. Black plastic bags are often similar to any dark portions in the image. Packaging materials are often similar to the reflecting surface of the tracker hopper. Some clear candidates of plastic bags include color bags (blue, yellow, purple), coles bags and woolie bags. As a labeling schema, six type of plastic-bag candidates were considered to be annotated for bounding box detection. The plastic-bag candidates included coles bags, woolie bags, color bags, white bags, black bags and packaging material. Annotations were done using the labelImg tool and labels were saved in .xml format, which were converted to KITTI for training purposes (see Figure 1).

The plastic-bag contamination detection dataset was generated/curated following a number of standard steps. As a first step, the raw images captured by the camera installed on the waste collection truck were acquired from the Remondis repository. These raw images were then sorted manually to select the training candidates that included visible plastic-bag contamination. The sorted images were then annotated for the plastic-bag bounding boxes using the defined labeling criteria. The final annotated dataset was then converted to KITTI format and split into training and validation subsets for performance evaluation of trained computer vision models. The validation dataset consisted of the images that were not presented during the training process and were unseen to the model, and were used for the performance evaluation of the models. The final dataset consisted of 1125 samples (i.e., 968 for training, 157 for validation) with a total of 1851 bbox annotations (i.e., 1588 for training, 263 for validation).

## 4. Automated Plastic-Bag Contamination Detection System

**Figure 2:**
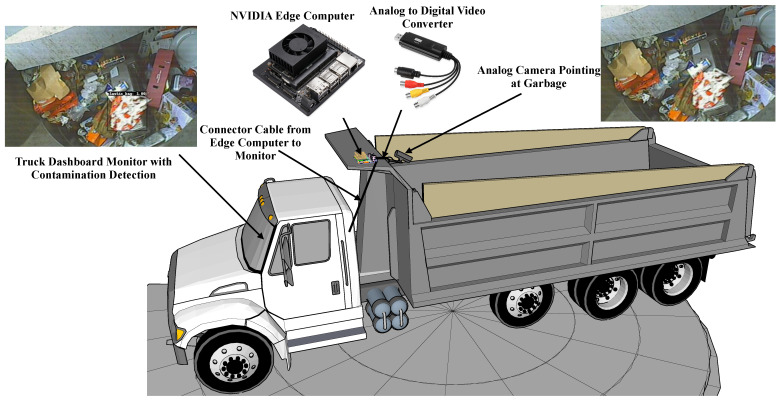
Conceptualization illustration of the proposed automated plastic-bag contamination detection.

To address the problem of detecting plastic-bag contamination in the waste collection trucks, an automated solution using edge-computing and computer vision approaches has been proposed. The concept of the proposed system is to make use of the already installed analog camera on the truck to process the images and deploy the latest computer vision models on edge-computing hardware to automatically detect plastic-bag contamination. The conceptual illustration of the proposed automated plastic-bag contamination detection system is shown in Figure 2. Overall, the system is designed to capture analog video from the installed camera, convert it to digital using the EasyCap analog-to-digital converter, make inference on a NVIDIA edge-computer using trained computer vision object detection models to detect plastic-bag contamination and display the detected contamination bboxes on the truck monitor. Fundamentally, the system uses trained computer vision models deployed on a NVIDIA edge-computer using the DeepStream application to process the input video feed towards detecting the plastic-bag contamination. Brief theoretical details about the computer vision object detection models and hardware components involved in developing the system are presented in the following subsections.

### 4.1. Computer Vision Models for Plastic-Bag Contamination Detection

Towards developing an optimized solution, as a Research and Development (R&D) approach, multiple variants of state-of-the-art computer vision object detection models (i.e., Faster R-CNN, YOLOv4) were trained and compared to identify the best performing model. The theoretical background to each of the implemented computer vision models is presented as follows.

#### 4.1.1. Faster R-CNN

**Figure 3:**
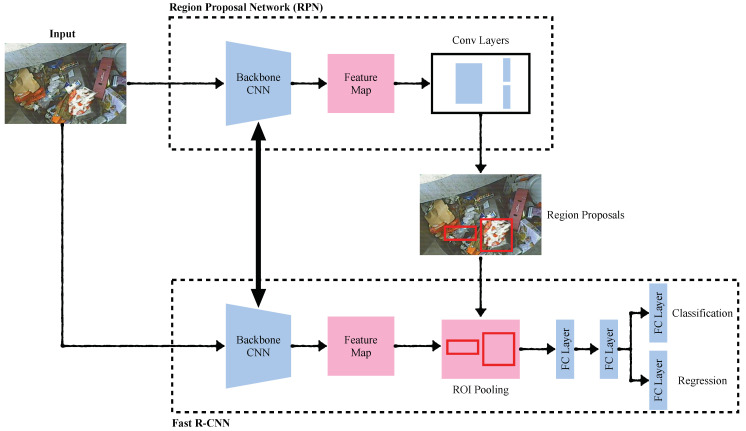
Overview of Faster R-CNN architecture.

The Faster R-CNN model was proposed by Ren et al. [30] and addressed the problem of high computational cost while calculating region proposals. This model is based on a novel Region Proposal Network (RPN) developed with the idea of sharing the features from the feature extraction network with the detection network, significantly reducing the computational cost. Further, the Fast R-CNN and RPN networks were merged using the shared CNN features and introduced the attention-based mechanism. In the RPN, anchors are used to address the multiple scales and aspect-ratio problems related to objects. As a result of this operation, an anchor is placed at the center of each spatial window. The proposals are then parametrized in relation to the anchors. This results in a unified single model with two modules: the RPN deep CNN model and the Fast R-CNN detector. Compared to other object detection models, the proposed RPN network generates multi-scale anchors as regression and adopts a pyramid type approach to make it efficient. Therefore, the loss function includes both the classification and regression tasks as expressed in Equation (1). It can be observed that both the regression loss and classification loss are optimized to train the model.

(1) $$L\left(\left\{p_{i}\right\},\left\{t_{i}\right\}\right)=\frac{1}{N_{\mathrm{cls}}}\sum_{i}L_{\mathrm{cls}}\left(p_{i},p_{i}^{*}\right)+\lambda \frac{1}{N_{\mathrm{bbox}}}\sum_{i}p_{i}^{*}L_{\mathrm{bbox}}\left(t_{i},t_{i}^{*}\right)$$

where *i* is the index for anchor, $p_{i}$ is the probability for the *i*th anchor, $p_{i}^{*}$ is the ground truth for the *i*th anchor, $t_{i}$ is the vector containing the predicted bbox coordinates, $t_{i}^{*}$ is the vector for the ground truth bbox coordinates, $N_{\mathrm{cls}}$ and $N_{\mathrm{bbox}}$ are regularization terms and λ is the balancing parameter. Figure 3 shows the architecture of the Faster R-CNN model.

In the process of training, first, the shared convolutional layers in the backbone network extract the deep features related to plastic-bag contamination from the images. This network is often referred to as the feature extractor. A fixed size image is selected as an input to the pooling layer along with the information extracted by the RPN layer. At the final stage, an output detection network with fully connected layers is connected with a high dimensional feature vector. One fully connected layer in the output network is used for the classification score determination, while the other layer is used for the position of detection by regression. The parameters of the neural network during the training process are adjusted based on the loss function (see Equation (1)). Given the output of the loss function, the SGD optimizer adjusts the weights of the network to minimize the loss of the model during the backpropagation process. This process takes place in the following steps:

- First, based on the backbone network, the weights (*w*) and bias (*b*) of the network are initialized;
- A forward-propagation process starts, which performs the computations on the input image based on the type of layer in the network.
  – For a fully connected layer, forward computation is performed using the following expression:
    $$\begin{array}{c} \alpha^{m,l}=\sigma \left(z^{m,l}\right)=\sigma \left(W^{l}\alpha^{m,l-1}+b^{l}\right) \end{array}$$
    where *m* denotes the image sample, *l* denotes the layer of the network, σ denotes the activation function (i.e., ReLU for this case), *W* denotes the network weights and *b* denotes the network bias;
  – For a convolutional layer, forward computation is performed using the following expression:
    $$\begin{array}{c} \alpha^{m,l}=\sigma \left(z^{m,l}\right)=\sigma \left(W^{l}⊗\alpha^{m,l-1}+b^{l}\right) \end{array}$$
    where ⊗ denotes the convolution operation;
  – For the pooling layer, a reduced dimension operation is performed on the input;
  – For the output layer, a Softmax function is used to predict the class probabilities. Softmax operation can be mathematically represented as:
    $$\begin{array}{c} \mathrm{Softmax}{\left(z\right)}_{j}=\frac{\exp^{z_{j}}}{\sum_{k=1}^{K}e^{z_{k}}}\;\mathrm{for}\;j=1,2,\cdots ,K \end{array}$$
    where *K* denotes the dimension of the *z* vector on which Softmax is being applied;
- Based on the loss function, a backpropagation operation is performed depending on the type of layer in network. The backpropagation process involves loss minimization using the gradient descent approach where weights and bias values are updated for each layer depending on the gradient values. Learning rate plays a vital role in the gradient descent process and has to be chosen carefully during the training process. For the Faster R-CNN training, a learning rate of 0.02 was used.

#### 4.1.2. You Only Look Once version 4 (YOLOv4)

The YOLOv4 model was proposed by Bochkovskiy et al. [31] with the aim to achieve accurate and high-speed performance for mobile platforms deployed in the field for real-time applications. Often, YOLOv4 is also referred to as an updated version of YOLOv3 with improved speed and accuracy. A number of universal features were introduced in the new model to be used for improved performance, including Cross-Mini-Batch Normalizations (CmBN), Cross-Stage Partial Connections (CSP), mish activation and Self Adversarial Training (SAT). The overall structure of YOLOv4 consists of an optimized backbone architecture, a neck architecture, and a detection head architecture. With default settings, YOLOv4 was developed using CSPDarkNet53 as a backbone, an additional SSP module, a PANet neck model, and a YOLOv3 head model. The CSPDarkNet53 backbone network divides the input into two parts; one part is passed through the DenseNet network, while the other part bypasses the network. The SPP and PAN are used mainly because of their enhanced receptive fields. In order to avoid the limitation of the fixed size input, a max pooling operation is performed at the SPP layer, which results in fixed output representations. To preserve the spatial information, PANet performs the pooling operation at multiple layer levels within the network. Finally, for the detection and localization of the objects, YOLOv3 head architecture is used.

**Figure 4:**
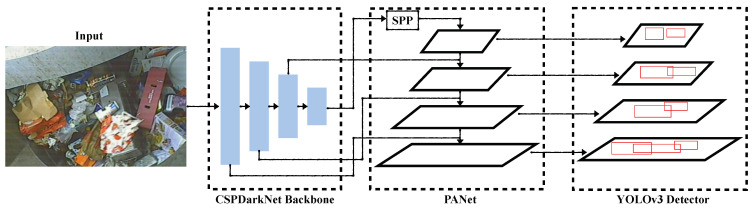
Overview of YOLOv4 architecture.

In terms of training performance enhancements, YOLOv4 introduced SAT and mosaic data augmentation approaches and uses genetic algorithms to optimize the model hyperparameters. The mosaic data augmentation approach mixes four training samples, eliminates the need for a large number of mini-batches and provides improved object features. On the other hand, in the SAT augmentation, the training image is modified and the model is trained on the modified image to detect objects of interest. The architecture of the YOLOv4 model is shown in Figure 4. The new YOLOv4 model outperformed the YOLOv3 model while keeping the real-time performance.

### 4.2. Hardware Components

**Figure 5:**
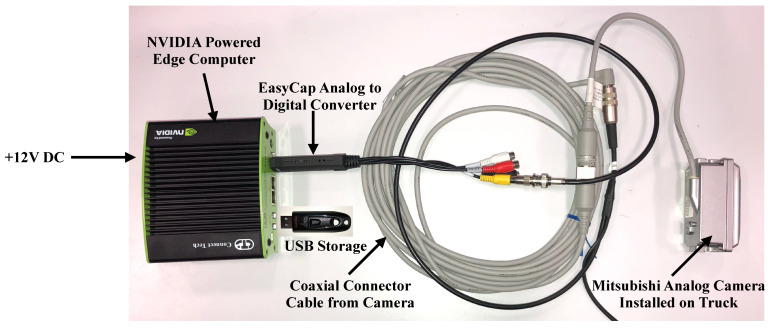
Laboratory hardware setup for the proposed automated plastic-bag contamination detection.

The proposed plastic-bag contamination system mainly consists of three hardware components: (a) a camera to capture the video, (b) an analog-to-digital converter, and (c) an edge-computer to process the video through the computer vision models to detect contamination. For the developed prototype, a Mitsubishi 4010 series analog video camera, an EasyCap analog-to-digital converter and a NVIDIA edge-computer were used. Figure 5 shows the laboratory hardware setup for the proposed plastic-bag contamination system. Brief details of each hardware component are provided as follows:

- Mitsubishi Analog Camera: Remondis waste collection trucks are already equipped with aluminum-encased Mitsubishi C4010 heavy-duty waterproof analog cameras specifically built for such harsh industrial utilities. The camera is capable of operating in low-lighting conditions and a high-vibration environment. The camera operates on +12V DC with 150mA current consumption and +50 ${}^{\circ }$C maximum operating temperature;
- EasyCap Analog-to-Digital Converter: To convert the analog video coming from the camera into digital for processing, an EasyCap USB 2.0 capture card was used. The capture card is a plug-and-play solution and supports high-resolution NTSC and PAL50 video formats;
- NVIDIA edge-computer: The edge-computer is the most important hardware component of the proposed system, with the role of performing all the computations related to plastic-bag contamination detection. For the developed prototype, NVIDIA Jetson Nano and NVIDIA Jetson TX2 edge-computers were used. The detailed specifications for both the edge-computers are presented in Table 2.

### 4.3. Experimental Design

To develop and validate the edge-computing solution for plastic-bag contamination detection, three experiments were performed:

- In first experiment, a variety of computer vision object detection models were trained and compared for their performance in detecting the plastic-bag contamination;
- In second experiment, the computer vision models were exported and deployed on the edge-computing hardware using a DeepStream video analytics application. The hardware performance of the models was compared for their suitability as a practical solution;
- In third and final experiment, the edge-computing hardware was deployed on three waste collection trucks where functionality of the developed solution was validated and additional data was collected. The collected data was then used to retrain the computer vision models for improved plastic-bag contamination detection performance.

## 5. Experimental Protocols and Evaluation Measures

**Figure 6:**
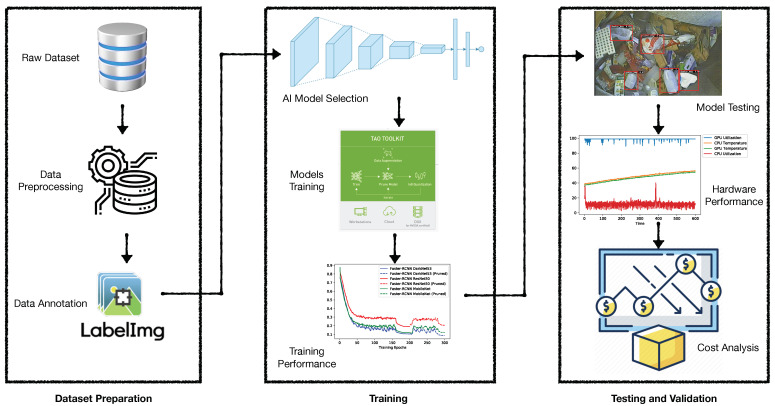
Block diagram representation of the research approach for automated plastic-bag contamination detection.

A standard three-stage data-driven research approach has been used for the development of an automated plastic-bag contamination detection system (see Figure 6). The first stage is referred to as the data preparation stage, where raw images collected from the Remondis records were sorted, filtered and processed. Further, at this stage, images were annotated using the LabelImg [32] annotation tool for the plastic-bag bboxes. The labels were converted to KITTI format to meet the requirements of the training platform. The second stage is referred to as the model training phase, where, first, the computer vision models were selected, taking literature as reference (i.e., Faster R-CNN, YOLOv4) and hyperparameters for training were decided. The NVIDIA TAO toolkit was used to train the selected models and training performance was assessed using the training loss, validation loss and validation mAP values to ensure that training followed the standard patterns. The final stage is referred to as the testing and validation stage, where the trained models were tested and evaluated using multiple software and hardware performance matrices. Furthermore, the detailed cost analysis was also presented at this stage to demonstrate usability for real-world application.

All the computer vision object detection models used in this research were trained using the NVIDIA TAO toolkit with TensorFlow and Python at the back-end. A NVIDIA A100 Graphical Processing Unit (GPU)-powered Linux machine was used to train the models. A data split of 80:20 was used for training and validation purposes, respectively. The Faster R-CNN model was trained using three different backbones (i.e., DarkNet53, ResNet50, MobileNet), while the YOLOv4 model was trained using two different backbones (i.e., CSPDarkNet53, CSPDarkNet_tiny). All the models were initially trained using a batch size of 1 for 200 epochs and were pruned (i.e., pruning threshold of 0.2 for Faster R-CNN models, pruning threshold of 0.1 for YOLOv4 models) and re-trained for 100 more epochs. Pruning is a commonly adopted approach in neural networks in which unnecessary connections between the neurons are removed to reduce the model complexity/size without impacting the overall model integrity. This results in achieving better memory usage, saving training time, and achieving faster inference times. However, the pruning threshold should be selected carefully since it is inversely proportional to the model prediction accuracy. A pruned model may observe a decrease in prediction accuracy mainly because some important weights might have been removed during the pruning process. Therefore, it is recommended to retrain the model after pruning to retain accuracy. For Faster R-CNN models, the Stochastic Gradient Descent (SGD) optimizer was used with 0.9 momentum and a base learning rate of 0.02 with L2 regularization. Multiple data augmentation techniques including scaling, contrast change and image flipping were incorporated into the training. For the YOLOv4 models, the Adaptive momentum (Adam) optimizer was used with L1 regularization and a base learning rate of $1\times 10^{-7}$. Image flip, color variations, and jitter data augmentation approaches were used during the training.

### Performance Evaluation Measures

The performance of the developed plastic-bag contamination detection system was assessed in terms of software and hardware using multiple matrices. The software performance was assessed in the training and testing phases separately. The training performance of computer vision models was evaluated using training loss, validation mAP, training time per epoch and monitoring of the training curves. The test performance of models was assessed using the mAP for the unseen validation dataset. The mathematical expression for calculating mAP is given in Equation (2).

(2) $$\mathrm{mAP}=\frac{1}{N}\sum_{i}^{N}{\mathrm{AP}}_{i}$$

where AP refers to the Average Precision, which is defined as the weighted sum of precisions at each threshold, where the weight equals the increase in recall. AP is determined from the precision-recall curve and is one of the most commonly used measures for evaluation of object detection model performance. *N* represents the number of classes. In this case, since there is only one detection class (i.e., plastic-bag), mAP is equivalent to AP.

The hardware performance of models was benchmarked using NVIDIA Jetson Nano and NVIDIA Jetson TX2 boards in terms of system usage (i.e., GPU usage, CPU usage, GPU temperature, CPU temperature), average power consumption and Frames Per Second (FPS). Finally, the cost analysis was reported to highlight the suitability for practical implementation of such a system towards automating the plastic-bag contamination detection process.

## 6. System Evaluation

This section presents the results of the developed plastic-bag contamination detection system subjected to software evaluation and hardware evaluation. Results are presented quantitatively, illustrated graphically and described qualitatively for each evaluation to highlight the important trends.

### 6.1. Software Evaluation

Computer vision models for plastic-bag contamination detection were evaluated for their training and testing performances. Quantitative results are presented in tabular form and graphical illustrations are presented as training curves.

#### 6.1.1. Training Performance

**Figure 7:**
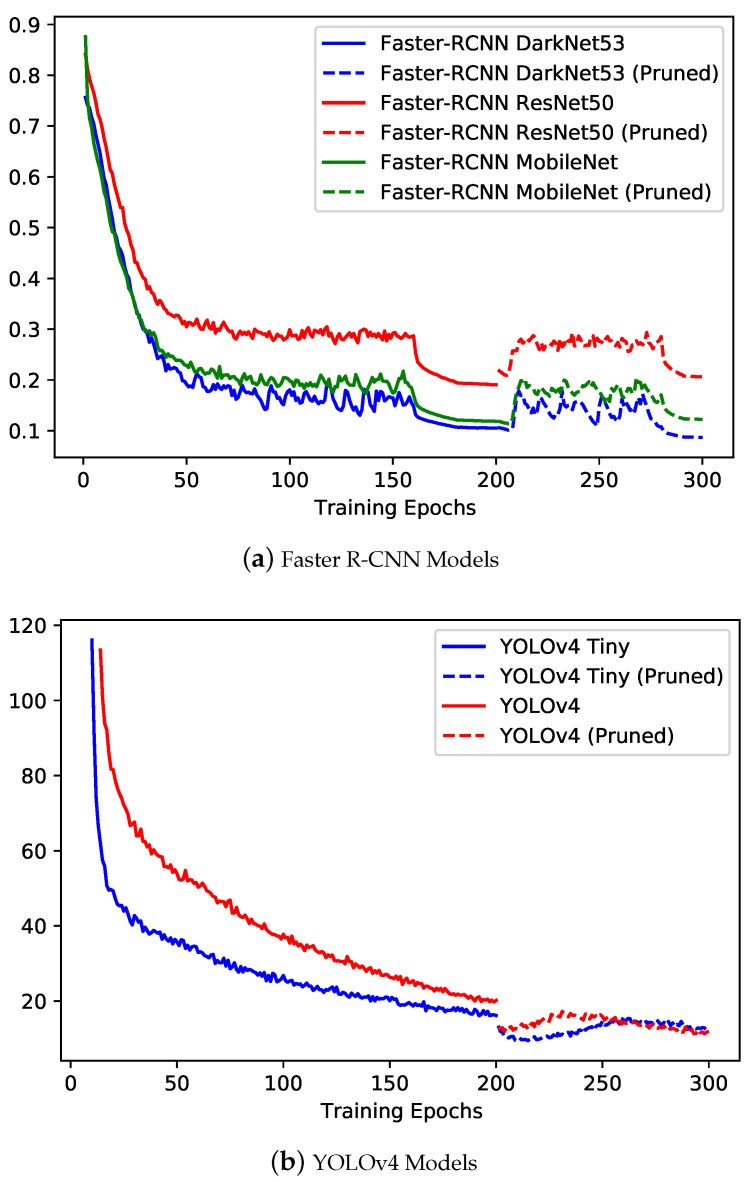
Training loss curves for the different variants of computer vision object detection models implemented for plastic-bag contamination Detection.

**Figure 8:**
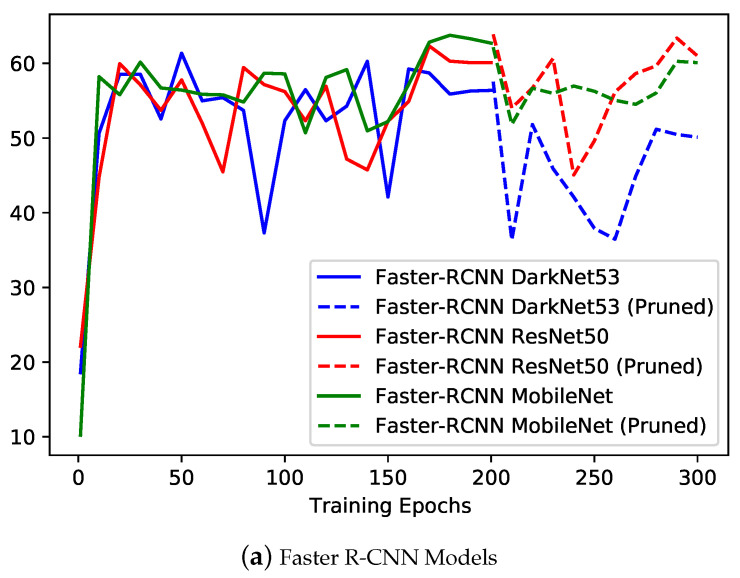

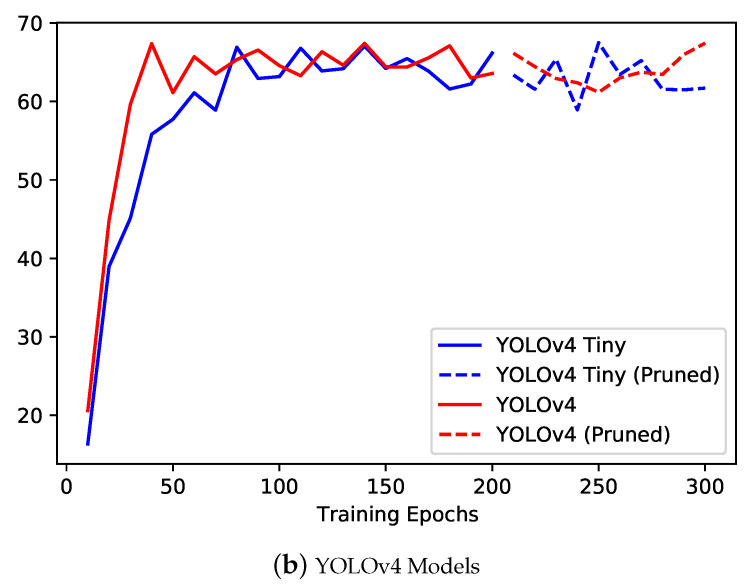
Training mAP curves for the different variants of computer vision object detection models implemented for plastic-bag contamination Detection.

The training performance was assessed using the training loss, validation mAP, loss curves, mAP curves and training time per epoch. The training loss curves and validation mAP curves for all the different variants of Faster R-CNN and YOLOv4 models are presented in Figure 7 and Figure 8, respectively. The curves for Faster R-CNN models and YOLOv4 models are presented separately because of the variation in the interpretation of loss for both types of models. For Faster R-CNN models (see Figure 7a and Figure 8a), it is observable that similar loss curve trend (i.e., negative exponential) was reported with DarkNet53 variant at the slight better end in comparison to MobileNet and ResNet50 variants. It can be observed that for all three models, after pruning, the loss increased for some epochs and then decreased to reach the minimum value. The degradation in the model accuracy was expected due to removal of important weights during the pruning process. However, upon retraining, the pruned model was able to retain the similar accuracy with much reduced model size (see Table 3 for model size comparison). The loss curves stabilized around 0.1 for DarkNet53 and MobileNet versions. However, from the mAP curves, it is observable that the MobileNet model and ResNet50 models achieved better performance in comparison to DarkNet53, specifically after the pruning of the model. ResNet50 and MobileNet models were able to achieve the maximum mAP of around 63% at the 290th and 190th epochs, respectively.

For YOLOv4 models (see Figure 7b and Figure 8b), a similar negative exponential trend was observed for training loss curves as in the case of Faster R-CNN; however, for YOLOv4 models, the loss kept on decreasing after pruning of models (i.e., evidence of effective pruning). Model pruning resulted in much reduced size model for YOLOv4 with CSPDarkNet in comparison to CSPDarkNet_tiny, for which very slight (i.e., negligible change) in size was observed (see Table 3 for model size comparison). YOLOv4 with the CSPDarkNet_tiny model performed slightly better in comparison to the CSPDarkNet53 variant, with loss stabilized around 18. From the mAP curves, comparatively similar performance can be observed, with the CSPDarkNet_tiny variant achieving a maximum mAP of 65% at the 170th epoch, while the CSPDarkNet53 variant achieving mAP of 67% at the 190th epoch.

The detailed impacts of pruning on computer vision detection models are quantitatively presented in Table 3. It can be observed that, for all the cases, pruning of models resulted in reduced model size, reduced training times and reduced number of trainable parameters. The training times are for relative comparison only and correspond to the training machine specified in experimental protocols section.

The detailed quantitative results from the training for the best performing epoch are tabulated in Table 4. The results are presented in terms of training loss, validation mAP, precision and recall score (i.e., for YOLOv4 models, precision and recall scores were not available). From Table 4, it can be clearly identified that the YOLOv4 model with CSPDarkNet53 backbone was able to achieve the best mAP of 67%, with a training loss of 21.83. The YOLOv4 with CSPDarkNet_tiny was reported second-best with slightly degraded performance (i.e., mAP of 65%).

**Figure 9:**
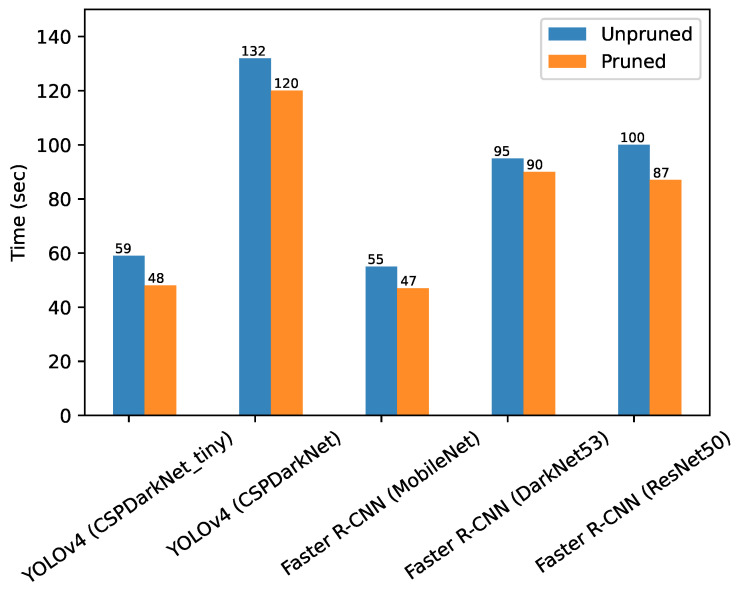
Training time per epoch for each implemented computer vision object detection model for plastic-bag contamination Detection.

Trained models were also evaluated for their relative training speed per epoch in seconds (see Figure 9) to determine the usability of training resources by each model. From Figure 9, it is evident that the YOLOv4 model with CSPDarkNet_tiny backbone was the fastest to train (i.e., 48 seconds per epoch), while Faster R-CNN with MobileNet backbone was second-best, with 55 seconds per epoch training time. The YOLOv4 model with CSPDarkNet53 backbone took the longest to train (i.e., 132 seconds per epoch), which may be attributable to the complexity of the model and the huge number of trainable parameters.

#### 6.1.2. Testing Performance

The trained computer vision models were subjected to an unseen validation dataset to evaluate their test performance (see Table 5 for detailed qualitative results). The test performance of implemented models was compared based on the mAP values. From the test results, the Faster R-CNN model with ResNet50 backbone was able to achieve an mAP of 64%, while YOLOv4 with CSPDarkNet_tiny backbone was able to achieve an mAP of 63%. The 64% mAP value for a single-class object detection problem is slightly on the lower end; however, it reflects the complexity and challenging nature of RCD. Given this, the performance of the best-performing model was observed to be comparable to the literature when a similar challenging real-world dataset has been used (i.e., 63.7% precision reported by Rad et al. [20], 78% mAP reported by Kraft et al. [26], 61% mAP reported by Patel et al. [27]).

### 6.2. Hardware Performance

**Figure 10:**
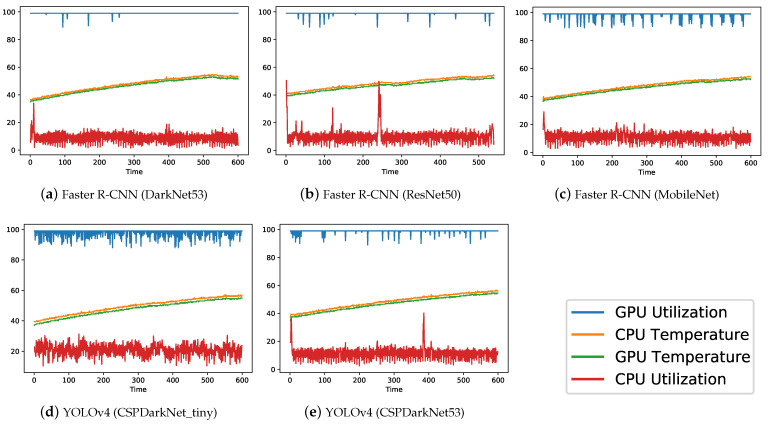
NVIDIA Jetson Nano system usage plots for different variants of implemented computer vision object detection models.

**Figure 11:**
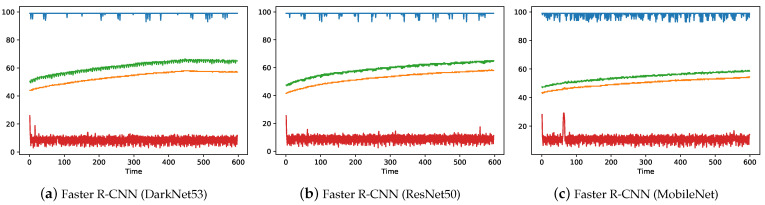

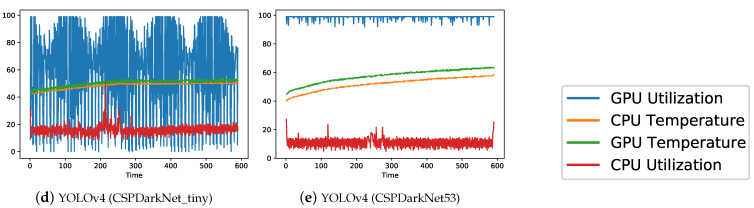
NVIDIA Jetson TX2 System usage plots for different variants of implemented computer vision object detection models.

The trained computer vision models were exported and benchmarked against NVIDIA Jetson Nano and NVIDIA Jetson TX2 edge-computers to compare their hardware performance in terms of system usage and power consumption. Results are presented in both tabular format (see Table 6) and illustrated graphically (see Figure 10 and Figure 11) to better compare the hardware performance of the implemented computer vision models. The performance was assessed based on FPS, average CPU usage, average GPU usage, maximum CPU temperature, maximum GPU temperature and average power consumption (available only for TX2). From the above-mentioned parameters, FPS, GPU usage and average power consumption are considered the most important factors in making the decision about which hardware and which model should be used for real-world deployment.

For the Jetson Nano board (see Table 6), it can be clearly observed that YOLOv4 with CSPDarkNet_tiny achieved the best performance in terms of FPS (i.e., 16.4), while the Faster R-CNN model with DarkNet53 was slowest, with only 0.4 FPS, mainly because of the complexity and depth of the model. For all the models (see Table 6 and Figure 10), GPU usage was observed to be the maximum (≈99%), CPU usage around 10% (except 21% for YOLOv4 with CSPDarkNet_tiny backbone), and temperatures stabilized to less than 60 degrees (i.e., within the operational temperature range referred in Table 2). The only model that can be used to achieve real-time performance in the real-world scenario using the Jetson Nano board is the YOLOv4 with the CSPDarkNet_tiny backbone.

For the TX2 board (see Table 6), a similar trend was observed as with the Nano board, where YOLOv4 with CSPDarkNet_tiny backbone was able to achieve the best FPS (i.e., 24.8), while Faster R-CNN with DarkNet53 backbone was slowest (i.e., 1.8 FPS). However, in contrast to Jetson Nano, for TX2, the Faster R-CNN model with MobileNet backbone and YOLOv4 with CSPDarkNet53 backbone models were also able to achieve higher FPS values of 8.4 and 6.6, respectively, making them suitable candidates for real-world application using the TX2 board. For all the models (see Table 6 and Figure 11), GPU usage was observed to be at maximum (≈99%) except for the YOLOv4 with CSPDarkNet_tiny backbone, where only 58.5% GPU was used. CPU usage around 10% (except 16% for YOLOv4 with CSPDarkNet_tiny backbone) and temperatures stabilized to less than 60 degree (i.e., within the operational temperature range referred in Table 2). In addition, for the TX2 board, average power consumption by each model was also recorded and, as expected, the YOLOv4 with CSPDarkNet_tiny backbone model consumed the least average power of 10.6 watts, in comparison to 16.9 watts consumed by the Faster R-CNN model with DarkNet53 backbone.

## 7. Discussion of the Results

**Figure 12:**
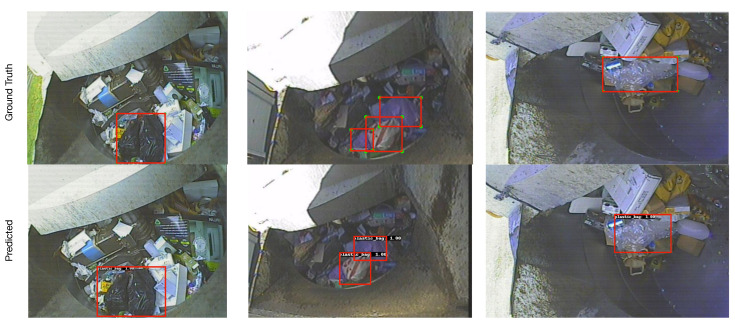
Sample correct predictions by the YOLOv4 with CSPDarkNet_tiny backbone model.

**Figure 13:**
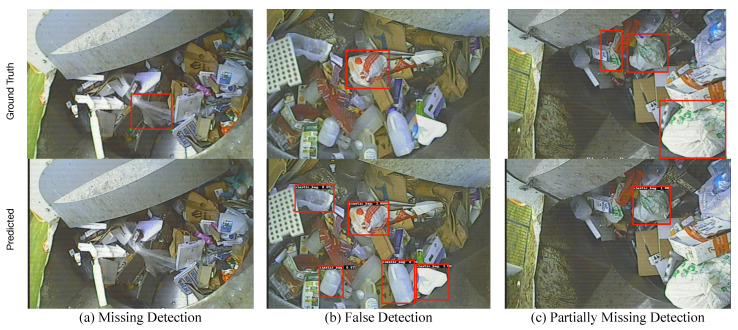
Sample false predictions by the YOLOv4 CSPDarkNet_tiny backbone model.

Results presented in Section 6 show that computer vision object detection models have considerable potential towards automating the process of detecting plastic-bag contamination in waste collection trucks. Furthermore, the hardware testing results further provided evidence that such models are practical to deploy in actual real-world scenarios. From the results, overall, the YOLOv4 model with CSPDarkNet_tiny backbone emerged as the most balanced model in terms of accuracy (i.e., 63%), speed (i.e., 24.8 FPS for Jetson TX2) and power consumption (i.e., 10.68 watts for TX2). Faster R-CNN model with MobileNet backbone and YOLOv4 model with CSPDarkNet backbone were also identified as potential second and third choices, respectively, for deployment using the TX2 edge-computer. Figure 12 and Figure 13 show true detection and false detection, respectively, for the YOLOv4 model with CSPDarkNet_tiny backbone. In Figure 12, it can be observed that the model was able to accurately detect the plastic-bag in the image, although the bboxes were not exactly the same as the ground truths; however, the model was able to capture the most of the plastic-bag in the image.

In terms of false detection (see Figure 13), three examples are included; first, when the model failed to detect any plastic-bag in the image; second, when the model wrongly classified other objects as plastic bags and third, when the model failed partially by detecting only a few of the many existing plastic bags in the image. One reason for the miss-detection may be attributed to the existing noise and visually similar objects within the dataset. However, it is expected that with the availability of more images for training, the model will keep improving and over a few iterations of re-training, it will achieve a level of accuracy acceptable for real-world application. The existing model has been deployed on actual waste trucks as a pilot project to test the functionality of the hardware and collect more images for fine-tuning the object detection model. A few highlighted challenges of the dataset identified from the analysis included the low pixel resolution of images (i.e., low level of visual details), presence of noise (i.e., light reflections, glare, low lighting) and visual similarity of the plastic-bag to other objects in the image (e.g., white bag similar to white boxes and white paper, black plastic-bag similar to the dark portions, packaging material similar to the shiny reflected surfaces).

## 8. Field Data Collection and Model Retraining

The developed edge-computing hardware was deployed in field for three waste trucks with the aim of validating the functionality of the developed solution and collecting more data. The DeepStream application was configured with the functionality to save the image and corresponding labels in KITTI format for each detection in an external USB drive. The idea behind this activity was to monitor the performance of the deployed model and to retrain the model using the collected data. From this activity, in total, 2325 images were extracted from the field deployment. Out of these image, 314 images were separated for testing, while 2011 images were used for the retraining of the model. In addition to images collected from the field, a set of images was also extracted from the open source videos captured by the waste collection truck. In total, 2224 images from the videos source were extracted and used for the retraining of the model. All the images were annotated for the plastic-bag bounding box instances.

The YOLOv4 model with CSPDarknet_tiny backbone (i.e., the best-performing base model reported in Section 7) was retrained with additional images collected from the field and extracted from the open source videos. In total, an additional of 4235 images were used along with the original 968 images for the retraining of the model towards achieving improved performance. The same experimental protocols as described in Section 5 were adopted for the retraining of YOLOv4 model with CSPDarknet_tiny backbone. From the retraining results, an improved performance of 73% mAP for YOLOv4 with CSPDarkNet_tiny backbone was achieved. In addition to training performance, to better monitor the improvement of the retrained model, both the base and retrained models were subjected to an unseen test dataset of 314 images collected from the field. The performance was compared in terms of mAP, True Positives (TP), False Positives (FP) and False Negatives (FN). Table 7 summarizes the field testing results for the base and retrained models. From the results, it can be observed that retrained model achieved mAP of 69% in comparison to the base model, which achieved mAP of 58% (i.e., an improvement of 11%). Furthermore, the number of FPs was observed to be reduced to 112 for the retrained model in comparison to 176 FPs for the base model (i.e., a reduction of 36.6% in the FPs). The FNs were also observed to be decreased by 8.29% for the retrained model. In addition, there was an increase of 6.21% in the TPs for the retrained model. The improved performance of retrained model suggests that a few more retraining iterations in the future using the data collected from the field will further improve the performance of the computer vision model for plastic-bag contamination detection.

## 9. Cost Analysis

Cost analysis for the developed edge-computing solution for plastic-bag contamination detection is presented in Table 8 to inform the stakeholders and define the baseline for deploying similar solutions in various geographical locations. The presented cost analysis is for the developed prototype based on the R&D principles and is subject to reduction by at least three times once the optimized version of the product is developed on a mass scale. Overall, the costs are divided into non-recurring costs (i.e., hardware cost, software cost, services cost) and recurring costs (i.e., software maintenance cost, hardware maintenance cost, operational cost). Non-recurring costs are estimated to be $22,245 (i.e., the hardware cost of $2245, software development cost of $15,000, the installation cost of $5000) and are to be spent one time. Recurring costs are estimated to be $15,225 for one year (i.e., the software maintenance cost of $10,000, hardware maintenance cost of $225, the operational cost of $5000).

## 10. Conclusions

An edge-computing video analytics solution has been successfully developed and validated for automated plastic-bag contamination detection in waste collection trucks. Multiple variants of the Faster R-CNN and YOLOv4 model were trained using real waste data collected from Remondis historical manual tagging records (i.e., RCD). From the results and analysis, in terms of training performance, the YOLOv4 model with CSPDarkNet53 backbone was able to achieve the best performance (i.e., validation mAP of 67%); however, it took the longest among all models to train (i.e., 132 seconds per training epoch). On the other hand, YOLOv4 with CSPDarkNet_tiny backbone was able to achieve a comparable training performance (i.e., mAP of 65%), but was the fastest to train (i.e., 48 seconds per training epoch). A similar trend was also observed for the testing, where the YOLOv4 model was the second best (i.e., 63% mAP in comparison to 64% for the best performing model). From a hardware deployment perspective, the YOLOv4 model with CSPDarkNet_tiny backbone was the fastest (i.e., FPS of 24.8 for TX2) and consumed the least power (i.e., 10.68 watts for TX2) in comparison to all the implemented models; therefore, it is suggested as the suitable model to be deployed on TX2 edge-computers for real-time plastic-bag contamination detection in waste collection trucks. The proposed edge-computing solution was deployed on waste collection trucks to assess the functionality of the system and to collect more data for model fine-tuning. As a result, around 4235 more images from the field testing and open source videos were collected, with which the YOLOv4 model with CSPDarkNet_tiny backbone was retrained for improved performance. The retrained model was able to achieve an improved performance in comparison to the base model in terms of mAP (11% increase), FP (36.6% decrease), TP (6.21% increase) and FN (8.29% decrease). For the proposed prototype development, $22245 USD is estimated for the one-time cost to deploy the system, while $15225 USD is estimated for per year recurring costs. The visual similarity of other objects to plastic bags was highlighted as one of the critical limitations in the presented research, along with low lighting conditions and the presence of reflections. In the future, it is planned to annotate images for multiple types of plastic bags (e.g., white bag, black bag, colored bag, coles bag, woolies bag) for improved performance. Furthermore, as an extension of this research, it is intended to make use of other cameras installed on the truck to detect potholes and roadside trash.

## Figures and Tables

**Table 1:** Summary of benchmark literature related to detection and classification of waste objects using computer vision techniques.

Author	Year	Addressed Problem	Dataset	Proposed Approach	Performance
Rad et al. [20]	2017	Litter classification	Custom dataset	OverFeatGoogleNet	Precision of
		and detection	(4000 images)		63.7%
Ibrahim et al. [21]	2019	Waste contamination	ContamiNet	CNN	AUC 0f
		detection	(30000 images)		0.88
Kumar et al. [22]	2020	Waste classification	Custom dataset	YOLOv3	mAP of
		and detection	(8000 images)		95%
Li et al. [23]	2020	Water surface	Custom dataset	YOLOv3	mAP of
		garbage detection	(1200 images)		91%
Panwar et al. [24]	2020	Water waste	AcquaVision	RetinaNet	mAP of
		detection	(369 images)		81%
White et al. [25]	2020	Waste object	TrashNet	WasteNet	Prediction accuracy
		classification	(2500 images)	((VGG)-16 based)	of 97%
Kraft et al. [26]	2021	Trash object	UAVVaste	YOLOv4, EfficientDet	YOLOv4 mAP
		detection	(774 images)	SSD	of 78%
Patel et al. [27]	2021	Garbage	Custom dataset	YOLOv5, EfficientDet	YOLOv5 mAP
		detection	(544 images)	RetinaNet, CenterNet	of 61%
Chazhoor et al. [28]	2022	Plastic waste	WaDaBa	AlexNet, ResNeXt, ResNet	ResNeXt AUC
		classification	(4000 images)	MobileNet, DenseNet	of 94.8%
Radzi et al. [29]	2022	Plastic waste	Custom dataset	ResNet50	Accuracy of
		classification	(2110 images)		94%
Ziouzios et al. [5]	2022	Waste detection	Custom dataset	YOLOv4	mAP of
		and classification	(4000 images)		92%

**Table 2:** Detailed hardware specifications of NVIDIA Jetson Nano and NVIDIA Jetson TX2 edge-computers.

	NVIDIA Jetson Nano	NVIDIA Jetson TX2
GPU	128-core NVIDIA Maxwell	256-core NVIDIA Pascal
CPU	Quad-core ARM A57	Quad-core ARM A57
Memory	2 GB 64-bit LPDDR4 25.6 GB/s	8 GB 128-bit LPDDR4 59.7 GB/s
Storage	16 GB eMMC 5.1	32 GB eMMC 5.1
Dimensions	69.6 mm × 45 mm	87 mm × 50 mm
Performance	472 GFLOPs	1.3 TFLOPs
Power	5/10 W	7.5/15 W
Temperature range	−25 ${}^{\circ }$C ∼ 80 ${}^{\circ }$C	−25 ${}^{\circ }$C ∼ 80 ${}^{\circ }$C

**Table 3:** Impact of pruning on the computer vision object detection models trained for the automated plastic-bag contamination detection.

Model	Model Size			Trainable Parameters			Training Time (per epoch)	
Name	Unpruned	Pruned		Unpruned	Pruned		Unpruned	Pruned
Faster R-CNN (DarkNet53 backbone)	344 MB	266 MB		42 M	33 M		95 s	90 s
Faster R-CNN (ResNet50 backbone)	342 MB	97 MB		42 M	24 M		100 s	87 s
Faster R-CNN (MobileNet backbone)	45 MB	24 MB		5 M	3 M		55 s	47 s
YOLOv4 (CSPDarkNet53 backbone)	594 MB	402 MB		49 M	38 M		132 s	120 s
YOLOv4 (CSPDarkNet_tiny backbone)	70 MB	69 MB		5 M	4.9 M		59 s	48 s

**Table 4:** Quantitative training results for the different variants of computer vision object detection models implemented for plastic-bag contamination detection.

Model	Training Loss	mAP	Precision	Recall
Faster R-CNN Models				
MobileNet backbone	0.1044	63%	0.4569	0.7425
DarkNet53 backbone	0.1915	61%	0.1448	0.8517
ResNet50 backbone	0.2106	63%	0.2961	0.7871
YOLOv4 Models				
CSPDarkNet53 backbone	21.83	67%	NA	NA
CSPDarkNet_tiny backbone	18.73	65%	NA	NA

**Table 5:** Quantitative testing results for the different variants of computer vision object detection models implemented for plastic-bag contamination detection.

Model	mAP
Faster R-CNN (DarkNet53 backbone)	50%
Faster R-CNN (ResNet50 backbone)	64%
Faster R-CNN (MobileNet backbone)	59%
YOLOv4 (CSPDarkNet53 backbone)	62%
YOLOv4 (CSPDarkNet_tiny backbone)	63%

**Table 6:** Quantitative hardware performance of implemented computer vision models benchmarked on NVIDIA edge-computers.

Model	FPS	Avg GPU	Avg CPU	Max CPU Temp	Max GPU Temp	Avg Power
		(%)	(%)	(C)	(C)	(watt)
**Jetson Nano 2GB**						
Faster-RCNN Models						
DarkNet53	0.4	98.945	9.435	54.5	53	NA
MobileNet	3.2	98.29	11.40	54.5	53	NA
ResNet50	0.88	98.82	10.42	54.5	52.5	NA
YOLOv4 Models						
CSPDarkNet_tiny	16.4	97.66	21.15	57	55	NA
CSPDarkNet53	2.4	98.70	11.63	56.5	54.5	NA
**Jetson TX2**						
Faster-RCNN Models						
DarkNet53	1.8	98.82	8.72	58	66.5	16.942
MobileNet	8.4	98.08	11.05	54.5	59	13.863
ResNet50	2.6	98.77	9.07	58.5	65	16.131
YOLOv4 Models						
CSPDarkNet_tiny	24.8	58.5	16.21	50.5	53.5	10.680
CSPDarkNet53	6.6	98.60	11.23	58.5	63.5	15.719

**Table 7:** The performance comparison of base model and retrained model on field collected test data.

	Base Model	Retrained Model	Percentage Change
mAP	58%	69%	11% Increased
False Positives (FP)	176	112	36.6% decreased
False Negatives (FN)	239	218	8.29% decreased
True Positives (TP)	338	359	6.21% increased

**Table 8:** Detailed cost analysis for proposed plastic-bag contamination detection system.

		Item	Quantity	Purpose	Cost (USD)
Non-Recurring One Time Cost	Hardware Cost	Mitsubishi analog camera	1	Capture the visual data from the hopper of the garbage truck.	≈100
		Analog-to-digital video converter	1	To convert the analog video feed into digital for processing through edge-computer.	≈20
		NVIDIA edge computer	1	Edge-computer to perform computer vision operations.	≈2000
		Power adapter	1	To power the edge-computer hardware.	≈100
		External USB storage	1	To store the contamination-detected images for future analysis.	≈25
	Software Cost	AI models development cost	NA	To train the AI models with the capability to process the raw data and extract waste contamination relevant information.	≈10,000
		Software Implementation	NA	To deploy the trained AI model(s) on the edge computing hardware.	≈5000
	Services Cost	Installation cost	NA	To visit potential sites and set up the hardware system.	≈5000
Recurring Cost	Software Maintenance	Tuning of computer-vision models	NA	To update and optimize the computer vision models and overall software firmware. A major part of listed price is anticipated cost for the AI model fine-tuning and performance improvements. The price is listed for twice-a-year updates.	≈10,000
	Hardware Maintenance	Replacement of the hardware components	NA	To manage the hardware components replacement and/or repair including camera, edge-computer, cables and USB drive. The anticipated life of hardware components is 10 years. The listed price is calculated relatively for one year.	≈225
	Operational Cost	Operations and logistics to maintain the hardware	NA	To perform the maintenance operations on site. This includes the labor cost and logistics. Listed is the price for twice-a-year maintenance operation.	≈5000

## Data Availability

Not applicable.

## Abbreviations

The following abbreviations are used in this manuscript:

COAG	Council of Australian Government
NSW	New South Wales
AIoT	Artificial Intelligence of Things
YOLO	You Only Look Once
RCD	Remondis Contamination Dataset
SSD	Single Shot Detector
RPN	Region Proposal Network
CmBN	Cross Mini Batch Normalizations
CSP	Cross Stage Partial Connections
SAT	Self Adversarial Training
GPU	Graphical Processing Unit
SGD	Stochastic Gradient Descent
Adam	Adaptive Momentum
FPS	Frames Per Second
mAP	Mean Average Precision
TP	True Positive
FP	False Positive
FN	False Negative
